# Hospital and Institutional News

**Published:** 1919-08-30

**Authors:** 


### HOSPITAL AND INSTITUTIONAL NEWS.

### EXPERIMENTS ON ANIMALS.

We learn that during 1918 in England and Scotland 23 new places, mostly military or municipal laboratories, were registered for the performance of experiments upon animals. Licences were held by 701 persons, and the returns show that of these 407 licencees performed no experiments. The total number of experiments was, however, 77,601, being 22,066 more than in 1917. Details prove that the majority of these were of such a nature that no exception could be taken except by those who are opposed in principle to any experiments whatever being performed on living animals. Under Group A are found 1,557 none of which are permitted without anæsthesia, and in 951 of them the animals remained under the influence of the anæsthetic until they were killed. Group B contains the vast majority, namely 76,053 which required no anæsthetic, being simple inoculations, hypodermic injections, feeding and similar proceedings. The purposes for which these were undertaken are in outline: 6,027 were done in the course of cancer investigation, 29,000 on behalf of Governmental departments, &c., and over 37,000 for the preparation of sera, vaccines and drugs. Inspectors frequently visited all the registered places without warning, and found all the animals satisfactorily cared for, and those holding licences attentive to the provisions of the Act.

### AT AN INFANT WELFARE CLINIC.

An unfortunate situation has developed at Edmonton, where the local maternity and child welfare centre is threatened with a medical boycott because the supervisor, Dr. Lawrence, has been dismissed by the District Council without (it is stated) giving him any reason for the dismissal. Protests have been received from the Edmonton Medical Society, the British Medical Association, and the National Association of Local Government Officers; while the representative of the Midwives' Association Committee has resigned her post as a protest against the Council's action. The Ministry of Health are also said to have written for an explanation, which the Council have appointed a special committee to supply. Without information as to the causes of dispute, it is impossible to pronounce any opinion on its merits. Obviously full publicity of the whole of the circumstances is required, in the interests not only of Dr. Lawrence, but of the District Council as well.

### (?) THE RETURN OF INFLUENZA.

We read that a liner recently arrived in Plymouth from Bombay with, among the thousand troops on board, five hundred cases of influenza and pneumonia. The figure appears almost unbelievably high, but probably many are cases of relatively slight illness, although the six deaths recorded suggest from our past experience that over a hundred men were probably seriously and constitutionally affected. The epidemic first broke out in the Mediterranean, and although the men have been segregated at a West-country rest camp with full precautionary measures and the matter is considered in safe hands, yet the occurrence reminds one that we are by no means *free* from the past year's influenza menace.

### BEER AND THE DRUG HABIT.

At a recent inquest on a death due to the drug habit, Dr. Waldo, H.M. Coroner for the City of London, in summing up to the jury, said that since the war, with the stress, nervous strain, and unrest accompanying it, several cases of drug-takers have come before him in which there has been a strong suspicion that the scarcity, bad quality, and high price of alcoholic drinks in the form of beer, wine, and spirits directly—or indirectly—led to the taking, as a substitute, of opium and its tincture known as laudanum, morphine, and heroin. The coroner said every encouragement ought to be given to the production and sale at moderate prices of light lager beer and wines of low alcoholic strength, for the use of manual and brain workers. Twelve shillings a dozen, as at present asked for as many pints of beer, is, he thinks, exorbitant, and outside the reach of the ordinary individual. Clarets, too, are much dearer, and scarcer still. Whilst most medical men agree that alcohol as a medicine is of the highest value, evidence of the evil effects of the custom of "nipping" spirits between meals is frequently made manifest in the coroner's court. Such abuse leads to disease of the brain, liver, and blood-vessels generally, followed too often by death. One of the sequels of the war noticeable is the growing habit of the substitution of methylated spirits in place of the dearer and less easily obtainable gin or whisky. The deleterious effects accruing from this vicious custom—moral, physical, and mental—is only exceeded by the more deadly poisons such as opium and its derivatives, and of cocaine. He emphasised the need for still greater and permanent restrictions in connection with the sale and disposal of poisons such as opium and cocaine.

### PROPOSED PORT RESTRICTIONS.

The proposed new regulations concerning the admission of undesirable persons arriving from foreign ports have been considered by the Association of Port Sanitary Authorities. The effect of their adoption would be that if a ship-master brought to this country verminous persons, he would have to take them back again. The Association wishes the regulations to be amended so that passengers shall be compelled to submit themselves to medical examination; that we should follow America in returning to the port of embarkation patients suffering from trachoma and allied diseases; and that port medical officers should be allowed some discretion in sending patients with infectious diseases to hospitals, because malaria and pneumonia have been added to the list of notifiable diseases. The Ministry of Health is to be asked to receive a deputation from the Association on the subject.

### DOCTORS' INCREASED FEES.

The matter of raising professional charges has been taken up by the British Medical Council, and it is thought that by October fees will have advanced by fifty per cent. above pre-war rates. Not only has the cost of living increased but also the cost of drugs and surgical instruments. Petrol is a great consideration, and the Association considers that only a fifty per cent. increase as a minimum will maintain doctors in anything like their pre-war status. Some months ago the local authorities were circularised asking that their medical salaries should be increased by one-third, and at that time doctors in certain districts correspondingly increased their private fees, but it is now in view of general representations that the matter is being taken up. Panel practitioners are now receiving a bonus of thirty to fifteen per cent. on their panel patients, but the B.M.A. is at present trying to negotiate with the Government with a view to adopting a new scale for next year.

### A LOCAL DECLINE IN TUBERCULOSIS.

The indications of an increase in tuberculosis throughout the country is said to be due principally to the relaxation of the preventive measures during the war. It is a relief, therefore, to find that in thickly populated Southwark there has been a diminution in the number of cases under treatment and notified. Dr. Horace Wilson, tuberculosis officer, in his annual report, states that 1918 records a marked improvement. He hopes that the constant increase of newly notified cases, which had been discouraging during the past three years, may now be succeeded by a fall. It is the notification of new cases which is significant. Dr. Wilson points out that, providing the number of new cases is stationary or declining, a high death-rate is not undesirable, because it means the removal of sources of infection. At the close of the year 669 patients were still attending the dispensary.

### CONSUMPTIVES AND CARE COMMITTEES.

Dr. Wilson pays a high compliment to the assistance given to him by his Care Committee. It is composed of representatives of various organisations, who assess the amounts payable for children sent away to institutions after carefully considering the financial position of the family, and, as far as possible, endeavour to find work through suitable channels for the adult wage-earners who have returned from sanatoria. This is still a serious problem, Dr. Wilson states, as it is hard for the man whose disease has been partly arrested to realise that his working value is not so great as that of a man in full health. He finds it difficult to obtain work under what he considers remunerative conditions, in addition to which his opportunity is limited by his desire for outdoor work. For these cases Dr. Horace Wilson welcomes the proposed extension of the labour colony system. With regard to discharged soldiers, of whom seventy-three were under treatment during the year, Dr. Wilson says that the Pension Committee had done splendid work for them, and spared neither trouble nor expense.

### HOMES FOR CHRONIC CONSUMPTIVES.

Recently The Hospital was able to publish an interesting official report prepared by Southwark Borough Council officials showing the powers possessed for securing the segregation of infectious cases of tuberculosis. Whilst some authorities are considering what powers they have, others are putting them in operation. Thus the Deptford Borough Council has arranged for what is termed a "home of rest" for advanced consumptives. Finding that the neighbouring borough of Greenwich has had the matter under consideration, Deptford concludes that it would be advantageous for the four adjoining boroughs—namely, Deptford, Greenwich, Lewisham, and Woolwich—to combine in providing accommodation, and so conferences are taking place. In spite of the dearth of housing accommodation, it has been discovered that four empty houses abutting on Greenwich Park can be rented at about £40 per annum each, and the scheme is to convert these houses into a home of rest for about eighty patients. It has been ascertained that the Insurance Committee will pay two guineas a week for every insured patient sent to the home, and that the L.C.C. will pay a similar sum in respect of non-insured cases.

### BRISTOL'S AMALGAMATION SCHEME.

A committee of the governing bodies and professional staffs of the British hospitals has been formed to consider an amalgamation scheme both of the administrative and medical work of the institutions. If the always great difficulties can be overcome, Bristol will possess a centralised hospital system, presumably on the lines which have been discussed at Birmingham. It is again urged that much waste of effort and money is involved in the existence of isolated hospitals, and also that the medical teaching of the city would greatly benefit. However, co-ordination is one thing and amalgamation another. There are often legal difficulties in the way of amalgamation from which co-ordination escapes. But in the past co-ordination has often been too loose and accomplished little; so that there are joint hospital wards whose work has been ill-defined and have hardly known for what purpose they existed. But the definition and co-ordination of medical services has much advanced of recent years. The creation of a Ministry of Health proves this, and with the impulse now set towards centralisation it should be possible to make the co-ordination of hospitals a living system, and where co-ordination is real and thorough amalgamation has very much less to say for itself.

### SHOP COLLECTIONS IN NORWICH.

Gradually, we are glad to note, the organisation of workmen's subscriptions is spreading from industrial centres to country and cathedral towns. Cambridge was early in the field with its district fund and shop collections, and the latest movement appears at Norwich. There a meeting of trades-union delegates was lately held to consider how to arrange collections from workshops and places that have hitherto not contributed to the hospitals at all. Some of the delegates were afraid lest the subscriptions of their unions should be interfered with, but they were asked merely to find a way of extending the existing funds. A delegate from the northern division of the Railwaymen's Union urged that employers and managers should organise a system of collections which would give additional hospital facilities to workmen. And it is true that the employers no less than the men must organise their generosity if the scheme is to be a success. It was also suggested that the recommendations should not be held by the firms, but by the secretaries of the trades unions. A committee was formed of those present to seek confirmation from the unions, and it was proposed that each factory in the city should form its own hospital and medical charities fund with its own officers. Each factory should then decide whether to base its subscriptions on a flat rate or according to the wages of the different men employed. The total should be sent to the secretary of the Hospital Saturday Fund in the name of the workmen of each factory, and that the fund have power to purchase any appliances needed by its members up to a certain sum each year. The smaller factories, it was suggested, should combine to purchase jointly the equipment. The plan promises well, and requires only a little energy to carry it out.

### THE SANCTITY OF DEEDS OF GIFTS.

A CURIOUS situation has arisen in connection with the Richard Murray Hospital at Blackhill, Yorkshire, which the Durham County Council is anxious to convert into a maternity and child-welfare centre. The permission of the Charity Commissioners must be joined before any change can be made, since the use of the buildings is limited by the trust deed, and the donor's intention, whatever it was, has not yet, it is stated, been adopted. Several local bodies have protested against the transfer, and it would be interesting to know why the donor's purpose has not been carried out. On general grounds, it is important that the wishes of the testators should be held sacred, because a man may be wiser than his heirs, and a wise man will be likely to endow a purpose to the value of which the fashion of time is blind. By protecting purpose of a trust, not only are we preventing its abuse, but doing what the law can to preserve after death the influence of those who may have been wiser than their fellows.

### SHADWELL PARK

THE problem of housing and the existence of parks and open spaces being both related to health, it is satisfactory to learn that the conversion of Shadwell Fish Market into a park in memory of King Edward is to be carried out. The site was purchased by the Mansion House Committee from the Corporation for £70,000, and during the war it has been used for military and Government purposes. The Committee, which needs £25,000 to complete the memorial scheme, expects to regain possession of the site before long, and East London will have, later or sooner, a new playground.

### HOLIDAY FUNDS—THE COLLECTORS' CHANCE.

THE holiday season is not supposed to be a productive one for hospitals, which postpone their appeals to the autumn. Nevertheless, there is for many hospitals an opportunity of raising money during the holidays which should not be missed—namely, by collections from visitors. Those hospitals in Cornwall, the Lakes, on the South and East Coasts, for example, should make use of the chance which comes in August to collect money from a class which cannot be approached at other seasons. It is the collectors' opportunity, and never, we learn, were holiday resorts so full as they are this year, nor were people, in spite of warnings, spending money more freely.

### ALLEGED VICTIMISATION OF V.A.D.s.

A CORRESPONDENT writes to complain of the grievance under which, he says, many V.A.D.s are suffering. The gist of the complaint is that young women who served as clerks and cooks do not receive the gratuities given on demobilisation to those who were employed as nurses. On the face of it this seems unfair, even if the authorities shelter themselves behind some technicality. At all events, the matter is not going to be allowed to rest, and will be raised in the House of Commons after the recess. Correspondents complain that the lay Press refuses to publish letters on the matter, perhaps (for we have not seen the rejected letters) because the plea for further gratuities would not strengthen the appeal for economy with which the silly season is being beguiled. While nurses cannot fail to note the prestige of their profession, to which the gratuity is granted as a matter of right, they will be public-spirited enough to wish to know on what grounds their fellow-workers are deprived of it. For after all it was an accident in most cases whether a young woman volunteered as cook, clerk, or nurse, and each department was necessary to the other.

### A PORTER'S DISMISSAL AND THE SEQUEL.

THE workmen's representatives at the half-yearly meeting of Merthyr General Hospital have declined to pass the report because the chairman, Mr. William Griffiths, would not permit a discussion on the dismissal of the porter. The porter was dismissed by the committee to which the matron had reported him after a dispute over the bread ration. The matter came before the committee, and, after full discussion, but by the casting vote of the chairman. the man was dismissed. In the meantime, it should be added, the matron had left the hospital. Under these circumstances the chairman saw no reason why the matter should be reopened. That the voting was so close suggests ample discussion, which it was his business, by his own vote, to determine. A casting vote, in our opinion, should be more sacred than any other, because its very *raison d'être* is to decide a much-disputed matter. Under these circumstances to rule a rediscussion out of order was the chairman's duty. When will working men realise that the end of administration is to put things through, not to discuss them interminably?

### A CURIOUS ADMINISTRATIVE SCHEME.

The allegations which led last November to the appointment of a Special Committee to inquire into the administration of the Greenwich Union Infirmary have been dismissed as unfounded. This decision was arrived at by a sub-committee appointed soon after the erection of a new ward last April. It was the business of this sub-committee to examine the lengthy correspondence which had accumulated. But the earlier report of the Special Committee deserves notice because of some curious recommendations that it contained. For instance, the Special Committee regarded the placing of medical and administrative control in the hands of a medical superintendent as a mistake. It preferred divided responsibility, and proposed that the medical superintendent should be responsible only for treatment, admission, and discharge; that the matron should have charge of nursing and domestic duties; that the fabric and supplies should be the responsibilities of the steward. Further, the Special Committee came to the conclusion that the medical superintendent should not live upon the premises. These proposals have the charm of originality, for we do not suppose that they would find favour in any other quarter, and it is, therefore, perhaps fortunate that the allegations which led to them have been dismissed.

### GUARDIANS AND A MEDICAL REFEREE.

Some discussion has been caused at the St. Pancras Board of Guardians by a request of the medical referee for superannuation purposes that his present fee of £1 1s. should be increased to £2 2s. After a proposal that the present fee be increased to 35s., which a member, Major Musday, described as insulting to the referee, Dr. Carr, the Guardians declined to raise his fee. The issues involved when a person claims to be entitled to superannuation allowance on the ground of ill-health are admittedly important; but a fee of one guinea for advising the Guardians about them seems to us quite ample, and it is difficult to support the proposal for doubling it.

### SUPERANNUATION ALLOWANCES AND INCREASED COST OF LIVING.

The Whitechapel Board of Guardians is inviting other Boards to support its proposal that the allowances of officials who have retired on superannuation should be increased to meet, in some degree, the increased cost of living. It appears that the Local Government Board, when appealed to, considered that there is no legal authority to increase amounts granted in the past. The Whitechapel Board now suggests that the superannuation allowances of all retired officials who receive £1 per week or less should be increased by 25 per cent., and appeals for support for this proposal. The depreciation in the value of money falls very hardly on many nurses and hospital servants who have retired on a small allowance, and in many cases real hardship is incurred at the present time. If bonuses are necessary for Government officials in active work to meet the increased cost of living, it is only fair that some additional grant should be made to a superannuation allowance the amount of which was fixed in relation to the then much greater purchasing power of money.

### STREET COLLECTIONS ON THE WANE.

As was expected by those who have had wide experience in the raising of funds for charitable purposes street collections, whether of the flag-day variety, or of that of the ordinary rattling of a money-box, are dying a natural death, and the statistics recently issued by Scotland Yard reveal that quite well-known charities received disappointing sums during the three months under review—April, May, and June of this year. The average expenses incurred in the collections was seven per cent., but in one case, that of the Kensington Philanthropic Society, the cost amounted to 30 per cent. of the total collected; we understand that organisations showing a heavy percentage of collection cost will not in future have an unconditional permit to renew the enterprise. The largest amount was collected by the "Our Day" Committee in aid of the Red Cross, when the gross proceeds amounted to £77,449, and the expenses to £4,838. In eleven cases representing gross receipts of £78,452 the expenses of administration amounted only to 10 per cent. or less, whereas in seven cases, yielding together £3,406, the gross cost of collection exceeded 15 per cent. was spent, and in two other ceeded 15 per cent. In the instance mentioned above 30 per cent. was spent, and in two other cases 20 per cent.

### BENEFACTIONS FOR BIRMINGHAM.

Under the will of the late Mr. Julius Jones, of Edgbaston, who died in June 1918, fifteen charities were recipients of legacies, and in May last a sum of £60,000 was distributed amongst them. As a result of further realisation of the estate we understand that a further £15,000 is available for distribution, and this additional allocation, it is stated, will come as an agreeble surprise to the institutions, which are as follows: The General Hospital, the Queen's Hospital (Birmingham), Birmingham and Midland Eye Hospital, Birmingham Royal Institution for the Blind, Birmingham and Midland Ear and Throat Hospital, Birmingham and Midland Skin and Urinary Hospital, Birmingham and Midland Hospital for Diseases of the Nervous System, Birmingham and Midland Hospital for Women, Birmingham and Midland Homœopathic Hospital and Dispensary, Royal Orthopædic and Spinal Hospital, Birmingham and Midland Free Hospital for Sick Children, St. Dunstan's Hostel for Blind Soldiers (London), Royal Orphanage (Wolverhampton), Walsall and District Hospital, Wolverhampton and Staffordshire General Hospital.

